# Fishing in the Dark: A Pursuit-Diving Seabird Modifies Foraging Behaviour in Response to Nocturnal Light Levels

**DOI:** 10.1371/journal.pone.0026763

**Published:** 2011-10-26

**Authors:** Paul M. Regular, April Hedd, William A. Montevecchi

**Affiliations:** Cognitive and Behavioural Ecology Program, Psychology Department, Memorial University, St. John's, Newfoundland and Labrador, Canada; Institute of Marine Research, Norway

## Abstract

Visual predators tend not to hunt during periods when efficiency is compromised by low light levels. Yet common murres, a species considered a diurnal visual predator, frequently dive at night. To study foraging of murres under different light conditions, we used a combination of archival tagging methods and astronomical models to assess relationships between diving behaviour and light availability. During diurnal and crepuscular periods, murres used a wide range of the water column (2–177 m), foraging across light intensities that spanned several orders of magnitude (10^3^–10^−10^ Wm^−2^). Through these periods, they readily dived under conditions equivalent to ambient moonlight (∼10^−4^ Wm^−2^) but rarely under conditions equivalent to starlight (∼10^−8^ Wm^−2^). At night, murres readily foraged during both moonlit and starlit periods, and diving depth and efficiency increased with nocturnal light intensity, suggesting that night diving is at least partially visually guided. Whether visually guided foraging is possible during starlit periods is less clear. Given the dense prey landscape available, random-walk simulations suggest that murres could benefit from random prey encounters. We hypothesise that murres foraging through starlit periods rely either on close-range visual or possibly nonvisual cues to acquire randomly encountered prey. This research highlights the flexibility of breeding common murres and raises questions about the strategies and mechanisms birds use to find prey under very low light conditions.

## Introduction

The foraging abilities and activity patterns of visual predators are strongly influenced by light levels [1]–[4]. Because prey detection depends on sufficient lighting, visual predators tend not to forage when efficiency is compromised by low light levels [5], [6]. In the marine environment, visually orienting predators contend with both temporal and spatial (i.e. depth) light restrictions. Such restrictions have contributed to the evolution of diel vertical migrations (DVM), whereby prey evade predation by residing in deep and dark waters during the day and move toward the surface at night [7]. Predation pressure has also favored the evolution of lunar cycles in DVM patterns of zooplankton [8]. Solar and lunar light availability therefore has pervasive effects on the foraging decisions of marine predators since it not only influences their ability to hunt visually, but also the vertical distribution of their prey [3], [6], [9].

To maximize foraging opportunity, many deep diving marine predators have evolved large, sensitive, dark adapted eyes [10]–[12]. The caveat is that visual adaptations for foraging at one light level generally compromises efficiency at another [13]. Owing to incompatible visual adaptations, species that forage through a wide range of light conditions likely experience the greatest visual constraints. Such species might even use non-visual cues to capture prey when foraging in conditions beyond their visual capabilities [14]. Indeed, it is not uncommon for marine predators to rely on alternate senses (e.g. tactile) to acquire prey through various conditions [15]–[18].

Common murres *Uria aalge*, the deepest diving flying species, are visually orienting pursuit-diving seabirds that in Newfoundland, Canada forage primarily on capelin *Mallotus villosus*, a DVM forage fish [19], [20]. To maximize overlap with their prey, breeding murres forage through diurnal, crepuscular and nocturnal periods and appear to adjust diving depths according to the DVM of capelin [21], [22]. These foraging patterns expose murres to low light levels through the diel cycle. Such activity may have selected for improved visual sensitivity in murres [13], though visually guided foraging may be ineffective under starlit conditions [23]. In this paper, we report on the use of a combination of archival tagging methods (temperature-depth recorders [TDRs] and temperature-depth-light recorders [TDLRs]) and astronomical models of light availability to investigate how light levels influence the foraging activities of murres.

## Materials and Methods

### Archival tagging

Archival TDRs and TDLRs were deployed on chick-rearing common murres during July and August, 2007–2010, at two Seabird Ecological Reserves in Newfoundland, Canada: Gull Island (47°16’N, 52°46’W), Witless Bay (∼100,000 breeding pairs in the reserve) and Funk Island (49°45’N, 53°11’W; 500,000+pairs [Canadian Wildlife Service unpublished data]). Adults were captured using a telescopic noose pole and equipped with an archival tag (Lotek Wireless, Canada; either TDR [LTD 1110 or LAT 1500] or TDLR [LAT 2500]). LTD 1110 s (5 g, 11×32 mm; 128 Kb memory) recorded internal device temperature (resolution±0.3°C) and pressure (depth resolution±0.49 m when maximum depth<125 m, and±0.98 m when maximum depth 125–250 m) every 2 s. LAT 1500 s (3.4 g, 8×35 mm; 512 Kb memory) recorded internal device temperature (resolution>0.05°C) and wet/dry state every 2 s, and pressure (0.05% resolution) every 2 s when the device was wet and depth>1.5 m. LAT 2500 s recorded the same parameters as the LAT 1500 s, in addition to light intensity (uncalibrated units) every 2 s when the device was wet and depth>1.5 m. TDRs and TDLRs were secured to plastic leg bands (Pro-Touch Engraving, Canada) and attached to the left legs of study birds; a Canadian Wildlife Service metal band was attached to the right leg. At Gull Island, 22 TDRs (n_2007_ = 6, n_2008_ = 11, n_2009_ = 5) and 17 TDLRs (n_2009_ = 9, n_2010_ = 8) were deployed on chick-rearing murres. At Funk Island, 30 TDRs (n_2007_ = 15, n_2008_ = 15) and 10 TDLRs (n_2009_ = 10) were deployed. Birds were typically recaptured after 3 days (range 2–7 days); three birds were recaptured the year following deployment. Birds were handled for ∼3 min and ∼6 min during logger deployment and recapture, respectively. 53 of 79 devices (67%), were recovered, of which 47 of 53 (87%) were successfully downloaded. Two records were excluded from analyses: one Gull Island bird that lost its chick and one Funk Island bird that was incubating. The analysis therefore included 13 TDR (n_2007_ = 3, n_2008_ = 5, n_2009_ = 5) and 12 TDLR (n_2009_ = 7, n_2010_ = 5) records from Gull Island, and 17 TDR (n_2007_ = 7, n_2008_ = 10) and 3 TDLR (n_2009_ = 3) records from Funk Island. Device memory lasted 36 h for LTD 1110 s and ∼150 h for LAT 1500 s and 2500 s. Records typically lasted for the duration of deployment.

Dives were considered submersions≥2 m since drift in the zero-level of TDRs and TDLRs exceeded±1 m in some cases. Using MT-Dive 4.0 (Jensen Software, Germany), the start and end time for each dive was determined, as well as the following parameters: maximum depth, bottom time and post-dive pause duration. Bottom time was defined as time elapsed between the first instance when vertical velocity dropped below 0.5 m s^−1^ and the last instance when it rose above 0.5 m s^−1^ [24]. Bouts were defined using maximum likelihood[25], and diving efficiency (bottom time/[dive+pause duration]) [26] was calculated for each bout.

Uncalibrated light readings from LAT 2500 TDLRs were calibrated to irradiance in Wm^−2^ using a photosynthetically active radiation (PAR; 400 to 700 nm) sensor (Biospherical Instruments, USA). Light level was varied for both devices through 32 synchronous deployments at water depths ranging from 0 to 290 m at sites between Funk Island and the northeast Newfoundland coast. TDLR sensor showed a strong log-linear response to PAR sensor irradiance readings, giving the following regression equation: output = 12.75*ln(irradiance)+349.04 (R^2^ = 0.97, p<0.0001). TDLRs were not sensitive enough to detect light when irradiance dropped below 10^−4^ Wm^−2^. This allowed the capture of complete light profiles for most dives, however light levels for nocturnal dives and the bottom portions of many twilight dives were not recorded. Minimum light intensity was directly calculated for dives with complete light records. For individual twilight dives with missing light data, light levels were predicted using dive specific attenuation values in those instances where sufficient numbers of light records (n>10) enabled detection of a trend. In other words, available light and depth data allowed the prediction of light levels experienced through the darkest portions of twilight dives. In such cases, predicted minimum light intensities were used. The absence of light records from nocturnal dives meant that minimum light intensies were not detected, nor could they be predicted. For these nocturnal dives, astronomical models were used to estimate light levels experienced.

### Astronomical model

For diving activity when in-situ light measurements were unavailable (TDR and night TDLR records), light levels were estimated using astronomical models. Similar to Zimmer et al. [3], sun angle (°) and absolute solar irradiance (Wm^−2^) for Gull and Funk Island were calculated using the formula in Iqbal [27]. Measures of moon angle, phase and absolute irradiance (Wm^−2^) were based on calculations in Jensen et al. [28]. Twilight light levels were not estimated because of a lack of available models. Global irradiance (light intensity at the water's surface) from the sun and moon was calculated after correcting for extinction of absolute irradiance from the earth's atmosphere and cloud cover. At St. John's, Newfoundland, Canada, global solar irradiance (measured at Memorial University of Newfoundland: http://www.physics.mun.ca/chemphysweather.html) was approximately 60%, 50% and 20% absolute solar irradiance during clear, partially cloudy and cloudy periods, respectively (cloud cover data from Environment Canada: http://www.climate.weatheroffice.gc.ca/Welcome_e.html). Spatially and temporally explicit estimates of light intensity were therefore calculated by applying appropriate percent extinction values, according to local cloud cover (data from nearest Environment Canada weather stations: St. John's for Witless Bay and Gander for Funk Island), to absolute solar and lunar irradiance. PAR at the surface was then approximated by multiplying total global irradiance by 50% [29]. To calculate underwater light intensity, an attenuation coefficient of −0.11 m^−1^ (mean attenuation experienced by TDLR equipped birds diving in Newfoundland water; sd = 0.06, n = 1687) was applied to the modeled surface light intensity values. Though there were significant differences in light attenuation experienced by TDLR birds at several levels (year, colony, individual, etc.), for modeling purposes, the mean value was used to estimate underwater light intensity. Overall, considerable natural variation was not incorporated into the model. This should not, however, limit our ability to detect general trends, especially since model results are used in conjunction with in-situ measurements.

### Statistics

Period by time of day was defined as follows: day was the period between sunrise and sunset (sun above 0°), dawn and dusk were when the sun was between 0° to –12° (nautical twilight), and night when the sun was below −12°. At night, moonlit and starlit periods were defined as occasions when the moon was above and below 0°, respectively. Lunar phase was defined according to the percentage of the surface visible: new 0–19%, crescent 20–39%, half 40–59%, gibbous 60–79% and full≥80%. We assessed depth and light utilization by examining diving depths and frequencies across periods. To test for light-related effects at night, mean hourly diving depths were regressed against mean hourly light intensities. To account for potential pseudoreplication, we used General Linear Mixed Models (GLMMs), fit by restricted maximum likelihood, with individual set as a random factor. *F*-tests assessed the significance of effects. For regressions, model fit was assessed using parameter estimate±95% upper and lower confidence intervals. All models were built and statistics run using R 2.10.1 [30]. Unless stated otherwise, means are presented with standard error values (mean±se).

## Results

**Figure 1:**
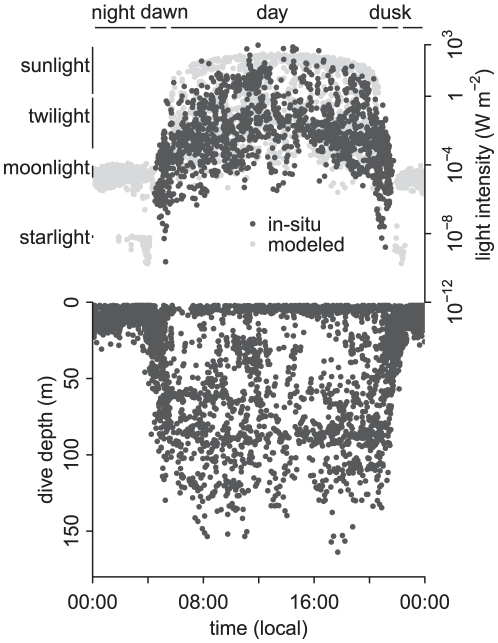
In-situ and modeled minimum light levels and associated maximum dive depths for 15 TDLR equipped murres across the 24 h period. Period duration and range of ambient light levels experienced from the sun, moon and stars are indicated.

**Figure 2:**
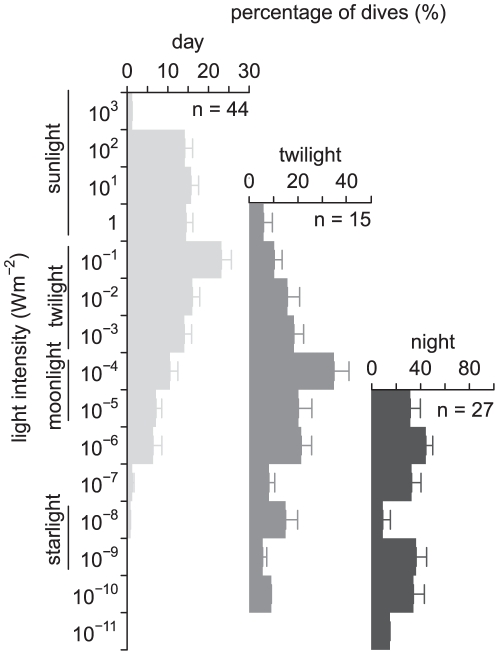
Histograms of light utilization during diving separated by period. Mean±se values across all individuals (n indicated). Daytime values are based on both in-situ and modeled light intensity whereas the twilight histogram is based solely on in-situ values and the night histogram was generated from modeled values (see methods). Lines left of plot give equivalent range of ambient light levels from the sun, moon and stars.

The diving behaviour described here is based on 9446 dives from 45 individuals across 114 recording days. Approximately half (4977) of these dives are from murres with TDLRs. By comparing model estimates of light intensities with in-situ measurements from TDLRs, it is apparent that modeled values are a good analog of actual light intensities experienced (figure 1). These data confirmed that light levels experienced by murres during foraging were highest during the day, rapidly declined through twilight and remained low at night (figure 1). Murres dive to extreme depths (max 177 m) only during daylight hours (figure 1). During the bottom phase of daytime dives birds frequently encountered light conditions equivalent to ambient twilight and moonlight levels (figures 1 and 2). It was very rare for murres to forage under light intensities equivalent to starlight during the day; light intensities remained>10^−8^ Wm^−2^ for 100±0.02% (range: 100–99%) of diurnal dives (figure 2). Diving depths and the light intensities experienced rapidly increased through dawn and decreased at dusk (figure 1). During twilight some foraging by TDLR birds occurred with lighting equivalent to starlight (figure 1), but almost all twilight dives (93±2.9%, range: 100–61%), were performed with light intensities>10^−8^ Wm^−2^(figure 2). Diving depths were restricted to<50 m at night (figure 1), when murres often foraged with<10^−8^ Wm^−2^ of available light; 56±8.3% (range: 100–0%) of nocturnal dives occurred in waters with lighting brighter than ambient starlight (figure 1). Therefore, while murres rarely forage under extremely dim conditions during diurnal and twilight periods, they readily forage under such conditions at night.

**Figure 3:**
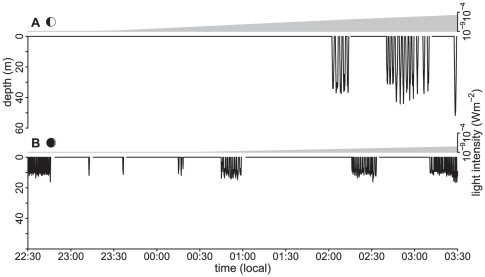
Changes in nocturnal diving behaviour, shown via dive profiles (black lines), in relation to modeled surface light intensity (grey area) from one murre diving during a half moon (a) and another during a crescent moon (b).

**Figure 4:**
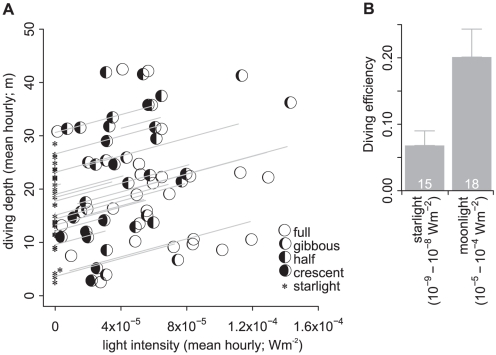
Mean hourly diving depth as a function of mean hourly light intensity (lines are GLMM fitted values by individual) (a) and diving efficiency under a moonlit versus a starlit sky (n indicated) (b).

Murres exhibit considerable variation in the nocturnal foraging behaviour. Some birds dived only when moonlight was available (figure 3*a*), others dived regardless of the availability of moonlight (figure 3*b*). Moreover, some individuals made different decisions on different nights. Such variation could be driven by a number of factors, such as colony specific constraints, individual differences, and/or location specific prey availability. But regardless of this variation, moonlight had a striking and consistent effect on murre diving behaviour (figure 4). Murres increased diving depth with increased lunar irradiance (GLMM: β = 8.4×10^4^ [LCI = 4.0×10^4^, UCI = 1.3×10^5^], F_1,69_ = 14.7, p = 0.0003; figure 4a). Diving efficiency was also significantly improved under a moonlit relative to a starlit sky (GLMM: F_1,229_ = 12.5, p = 0.0005; figure 4b). There were no annual or colony differences in these trends.

## Discussion

Murres dived through a broad range of light levels – from sunlit to starlit – as they foraged throughout the day and night. This is perplexing since it seems unlikely that their eyes could be adapted for visually guided foraging across all conditions [13], [31]. Previous research suggests that murres are adapted for diurnal and crepuscular hunting [21], [32], [33], implying that they are foraging at the limits of their visual abilities at night. It is important to consider, however, that deep diurnal and crepuscular diving frequently exposes murres' to conditions that match the light available from the moon. Though low light levels can constrain visual abilities and reduce foraging success [2], [6], [34], the propensity of murres to forage under such conditions suggests they are better suited for nocturnal diving than previously thought. Their diurnal and crepuscular foraging activities likely necessitate eyes optimized for visual sensitivity [12], [13]. Such visual adaptations would improve their ability to forage at night, especially when moonlight is available. The eye structure of murres may therefore share features of nocturnal birds. For example, king penguins *Aptenodytes patagonicus* – a pursuit-diving seabird which also forages throughout the diel cycle and experience similar light levels (10^3^–10^−4^ lux ≈1–10^−7^ W m^−2^ [34]) share similarities in axial length, corneal diameter and maximum pupil diameter with those of nocturnally active Tawny Owls *Strix aluco* [12].

Patterns in murre diving behaviour suggest that moonlight aids visual hunting at night. Light availability during moonlit periods matched conditions experienced during crepuscular and deep diurnal dives. Further, diving depths increase with increased nocturnal light levels. We therefore suspect that foraging is visually guided under moonlit skies. But what of foraging activity during starlit periods when light intensities drop below 10^−8^ W m^−2^? Murres rarely encounter such dark conditions during diurnal and crepuscular periods yet, contrary to expectations, they readily forage under these conditions at night. Reduced diving efficiency implies that there are behavioural consequences for foraging under starlit conditions. It is unknown whether murres have the spatial resolution necessary for visual foraging under starlight. Martin [12] suspects that king penguins lack the visual abilities to detect the outline of individual prey during starlit periods and considers the possibility that their nocturnal foraging activities are guided by the detection of light from the photophores of their prey[6], [12] cf [34]. Though prey with photophores, such as euphausiids and myctophids, have not been recorded in the diet of murres during the breeding season [19], [20], it is possible that murres switch to taking such prey under low light conditions. It seems more plausible, however, that breeding murres forage on capelin through the diel cycle since they feed almost exclusively on capelin [19], [20] and their diving patterns match capelin DVM [21]. Capelin do not have photophores thus murres are left to rely on ambient light for their detection. Though the murres' visual capacities are unknown, their ability to hunt visually is likely reduced under starlit conditions. If visual detection of prey occurs, it would seem possible only at close range [2], a constraint that would presumably greatly reduce foraging efficiency. Nevertheless, the fact that birds persistently dive during starlit periods suggests that their ability to capture prey is not completely compromised. How then is this possible?

**Figure 5:**
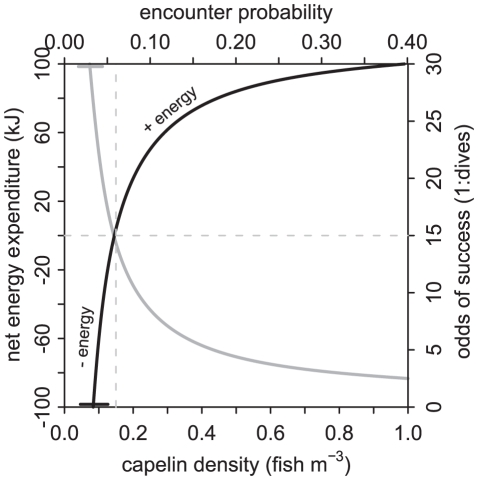
Modeled relationship between capelin density and net energy expenditure (black line). Net energy expenditure is a function of the number of dives required (grey line; energy cost) to encounter one capelin (energy gain). Results are based on 200 000 three-dimensional correlated random walk simulations.

In theory, murres foraging through starlit periods should be able capture prey they randomly encounter at close range. We explored the viability of this foraging strategy using correlated random walk simulations (File S1) and found that hunting through random encounters could be viable if prey were available in sufficiently high densities. Simulations suggested that birds would have to forage in capelin aggregations in excess of 0.15 fish m^−3^ in order to gain energy (figure 5). Capelin are not always this dense in Newfoundland waters; density depends on time and place [35]. However, capelin occur in coastal aggregations in densities exceeding 0.15 fish m^−3^ during their spawning period (Brian Nakashima, Fisheries and Oceans Canada, personal communications); and such densities have been recorded at night in shallow waters near Funk Island (Gail K. Davoren, University of Manitoba, unpublished data). In general, murres in the present study were rearing chicks during the capelin spawning period, but capelin are patchily distributed [36] and thus prey encounters would at least partially depend on being in the right location. Murres could decide whether to forage during starlight based on knowledge of patch quality gained earlier in the evening, as they tend not to fly/search at night (PMR unpublished observations). Such dynamics likely contributed to the variable nocturnal foraging patterns observed in this study.

The murres' nocturnal diving behaviour might also provide information about capelin vertical distribution. Increases in murre diving depths with heightened nocturnal light levels might indicate that capelin limit their vertical migration under these conditions. Galápagos fur seals *Arctocephalus galapagoensis* exhibit a similar response to lunar light, and this change was attributed to vertical shifts in prey distribution [9]. Murres track the diel vertical movements of capelin [21] and it is common for diel vertical migrants to adjust their vertical distribution at night according to lunar cycles [8]. Thus, perhaps murres dive deeper during moonlit periods to access prey located deeper in the water column.

In conclusion, we interpret our data to indicate that murres use moonlight to hunt visually at night but that they may switch foraging tactics when diving during starlit periods. If murres are able to visually detect prey under starlight, then the distance at which this is possible is likely to be greatly reduced relative to forging during moonlight. We hypothesise that murres foraging through starlit periods rely on close-range visual and/or non-visual cues to capture prey that are encountered randomly. Our research revealed aspects of a species' behavioural ecology which caused us to rethink their foraging abilities. Like several other deep diving marine predators [10]–[12], murres may indeed possess exceptionally sensitive eyes. In the same token, they may rely on alternate sensory cues when vision is constrained cf [15]–[18]. Though the physiological mechanisms behind the murres' ability to hunt through wide-ranging light conditions have yet to be understood, their ability to function through such conditions is a testament to their adaptability.

## Supporting Information

File S1 **Includes a description of the methods used for the correlated random walk model simulations, as well as a figure showing a sample simulation.**
(DOC)
