# Supplementary material for: Fishing in the Dark: A Pursuit-Diving Seabird Modifies Foraging Behaviour in Response to Nocturnal Light Levels
Source: PLoS One. 2011 Oct 26;6(10):e26763. doi: 10.1371/journal.pone.0026763 (PMC3202575; doi:10.1371/journal.pone.0026763)
Supplement: File S1 — Includes a description of the methods used for the correlated random walk model simulations, as well as a figure showing a sample simulation. (DOC) [file pone.0026763.s001.doc]

# File S1

## Model simulations

To test the viability of searching randomly for prey (i.e., foraging in the dark), we used a correlated random walk model to determine prey densities whereby the cost of encountering prey by chance would be offset by success. Capelin are the primary prey in this model because available evidence suggests that adult murres feed primarily on capelin during chick-rearing (stomach contents 99.6-100% capelin or unidentified fish [1,2]). Dives were simulated in three-dimensions using Gaussian distributed step-lengths and Wrapped-Cauchy distribution (WCD) turning angles [3]. Step-lengths were based on the mean±sd horizontal velocity of foraging common murres, 2.18m s­^-1^±0.43 [4]. The WCD turning angle parameter was set at 0.7, which resulted in dive profiles with ‘wiggles’ analogous to those observed in murres diving under starlight. Birds are presumed to be foraging during the bottom portion of dives [5], thus simulations were based on the average bottom depth and duration of murre dives during starlit periods. Capelin were randomly distributed in space since they form a scattered layer of individuals at night [6]. Encounters were considered instances where murres occupied the same space (±10cm) as one capelin during a dive. Encounter probability was calculated under capelin densities between 0.00001 and 1 fish m^-3^ (range in Newfoundland water [7]). 200 000 simulations were run (10 000 simulations at 20 capelin densities). The resultant probabilities were used to calculate the number of dives required to capture one capelin. To approximate net energy expenditure, energy obtained from a gravid female capelin (121kJ [8]) was subtracted from diving cost (8.34kJ; 7.8W kg^-1^ for resting and 14.2W kg^-1^ for diving metabolic rate [9] were used to approximate diving costs, including post surface pause, for an average starlit dive by an average-sized Funk Island murre).

## References

1. Piatt JF. (1987) Behavioural ecology of common murre and Atlantic puffin predation on capelin: Implications for population biology. St. John's: Ph.D. Thesis, Memorial University of Newfoundland.
2. Wilhelm SI, Robertson GJ, Taylor PA, Gilliland SG, Pinsent DL (2003) Stomach contents of breeding common murres caught in gillnets off Newfoundland. Waterbirds 26: 376-378.
3. Bartumeus F, Da Luz MGE, Viswanathan GM, Catalan J (2005) Animal search strategies: A quantitative random-walk analysis. Ecology 86: 3078-3087.
4. Swennen C, Duiven P (1991) Diving speed and food-size selection in common guillemots, *Uria aalge*. Neth J Sea Res 27: 191-196.
5. Halsey LG, Bost C-, Handrich Y (2007) A thorough and quantified method for classifying seabird diving behaviour. Polar Biol 30: 991-1004.
6. Davoren GK, Garthe S, Montevecchi WA, Benvenuti S (2010) Influence of prey behaviour and other predators on the foraging activities of a marine avian predator in a Low Arctic ecosystem. Mar Ecol Prog Ser 404: 275-287.
7. O'Driscoll RL, Rose GA, Anderson JT (2002) Counting capelin: A comparison of acoustic density and trawl catchability. ICES J Mar Sci 59: 1062-1071.
8. Montevecchi WA, Piatt J (1984) Composition and energy contents of mature inshore spawning capelin (*Mallotus villosus*) - Implications for seabird predators. Comp Biochem Phys A 78: 15-20.
9. Croll DA, Mclaren E (1993) Diving metabolism and thermoregulation in common and thick-billed murres. J Comp Physiol B Biochem Syst Environ Physiol 163: 160-166.

Figure S1. Three-dimensional correlated random walk simulation of a murre dive (black line) with capelin (grey points) occurring at a density of 0.1 fish m^-3^.
